# Improving mathematical modeling of interventions to prevent healthcare-associated infections by interrupting transmission or pathogens: How common modeling assumptions about colonized individuals impact intervention effectiveness estimates

**DOI:** 10.1371/journal.pone.0264344

**Published:** 2022-02-28

**Authors:** Camden D. Gowler, Rachel B. Slayton, Sujan C. Reddy, Justin J. O’Hagan

**Affiliations:** 1 Division of Healthcare Quality Promotion, Centers for Disease Control and Prevention, Atlanta, Georgia, United States of America; 2 Department of Ecology and Evolutionary Biology, University of Michigan, Ann Arbor, Michigan, United States of America; Johns Hopkins University, UNITED STATES

## Abstract

Mathematical models are used to gauge the impact of interventions for healthcare-associated infections. As with any analytic method, such models require many assumptions. Two common assumptions are that asymptomatically colonized individuals are more likely to be hospitalized and that they spend longer in the hospital per admission because of their colonization status. These assumptions have no biological basis and could impact the estimated effects of interventions in unintended ways. Therefore, we developed a model of methicillin-resistant *Staphylococcus aureus* transmission to explicitly evaluate the impact of these assumptions. We found that assuming that asymptomatically colonized individuals were more likely to be admitted to the hospital or spend longer in the hospital than uncolonized individuals biased results compared to a more realistic model that did not make either assumption. Results were heavily biased when estimating the impact of an intervention that directly reduced transmission in a hospital. In contrast, results were moderately biased when estimating the impact of an intervention that decolonized hospital patients. Our findings can inform choices modelers face when constructing models of healthcare-associated infection interventions and thereby improve their validity.

## Introduction

Healthcare-associated infections (HAIs) impose a substantial burden of disease and can increase healthcare costs considerably [1–5]. However, it is frequently challenging to estimate the effectiveness of prevention measures [6, 7]. Key barriers include the fact that there is generally a low incidence of infections in any one hospital, and therefore individual facilities have little opportunity to quantify the impact of an intervention in a short study. Multicenter studies have been conducted to help address this issue, but their cost can be prohibitive and the length of follow-up can be relatively short [8, 9]. Further complicating the problem, patients transfer between hospitals, nursing homes, other healthcare settings, and the broader community; thus, transmission and symptom onset in non-hospital settings can complicate efforts to evaluate the impacts of hospital-based interventions [7, 10–12].

To address such challenges, investigators sometimes use simulations to help assess whether a previous intervention was effective or whether a new intervention is likely to be successful [13, 14]. Transmission models are relatively inexpensive and quick to create, but require additional assumptions compared to traditional epidemiologic approaches (e.g., randomized trials, cohort studies) [15, 16]. Concerningly, even seemingly innocuous assumptions that are introduced to simplify an analysis have the potential to alter results in ways that modelers did not expect. For instance, modelers often simulate asymptomatically colonized individuals as having higher rates of hospital admission [17, 18] or longer lengths of hospital stay [19–35] compared to uncolonized individuals *because* they are asymptomatically colonized. While it is true that colonized individuals on average spend more time in hospitals per admission and are more readily admitted, there are likely several confounding factors that can explain the association [36]. For example, people asymptomatically colonized with an HAI causing pathogen tend to be older and sicker than non-carriers [37]. Consequently, a more plausible explanation is that people in poor health are more likely to be hospitalized and to have long lengths of stay, and thereby are more likely to become colonized with a HAI causing pathogen. Reverse causation can also explain some of the crude association between carriage status and length of stay [38]. As a result, transmission models could provide inaccurate estimates of intervention impact when asymptomatic carriage is simulated in these ways.

Therefore, our primary aim was to test whether the assumptions regarding higher rates of hospital admission or longer lengths of hospital stay for asymptomatic carriers can affect the results of simulations that evaluate the effects of interventions that work by preventing transmission of HAIs interventions.

## Methods

### General model description

**Fig 1:**
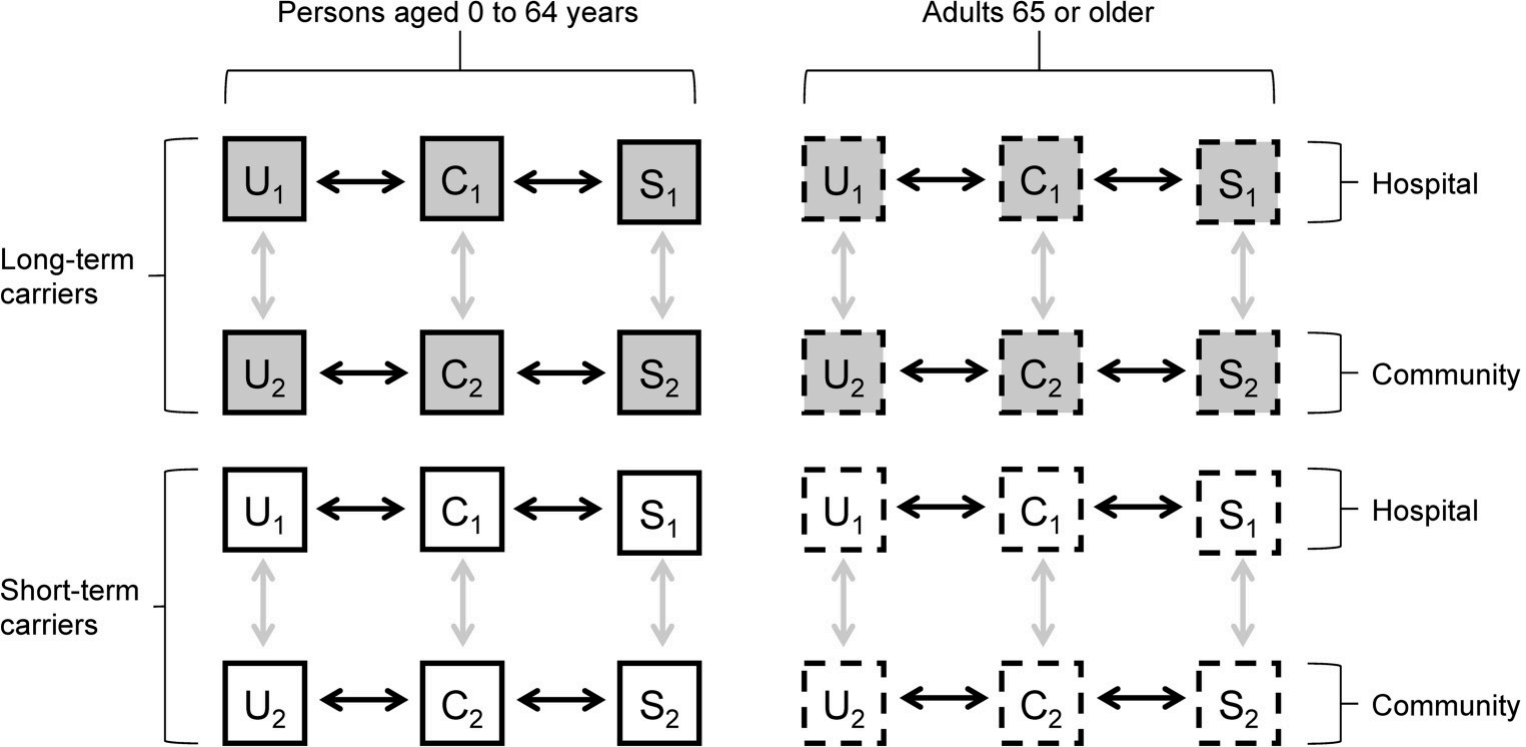
Flow diagram of general model. A flow diagram of the model with compartments for each combination of (i) Uncolonized (U), Colonized (C), or Symptomatic (S); (ii) persons aged 0 to 64 years or adults 65 or older; (iii) short-term or long-term carrier types; and (iv) subscript denoting location in a hospital (1) or community (2). Gray arrows show movement between the hospital and community, through admission and discharge, whereas black arrows show transmission, disease progression, recovery from disease, and loss of carriage. Solid and dashed borderlines denote compartments for each age strata, and gray shading denotes the long-term carrier compartments. Individuals could not move between age groups or carrier types. Full model equations are in S1 Appendix.

We created a deterministic compartmental model with Uncolonized, Colonized (asymptomatic carriers), and Symptomatic disease states to simulate transmission of an HAI pathogen in a hospital and the surrounding community (Fig 1). For each state, we included separate compartments for each combination of location (hospital or community), carrier type (short or long durations of carriage), and risk group. In our examples, we used two age groups (persons aged 0 to 64 years and adults 65 or older) to define the risk groups since older age is generally recognized as a proxy for increased rates of hospitalization and longer lengths of stay, and age-specific hospitalization data are widely available (Table 1) [39]. We parameterized the model so that adults 65 or older spent more time in the hospital than persons aged 0 to 64 years because of their higher admission rates and longer lengths of stay (Table 2). Individuals could move between the hospital and community but, to simplify the model, they could not transition between age groups or carrier types. We parameterized the model using data for methicillin-resistant *Staphylococcus aureus* (MRSA, Table 2, [29, 40]). Short-term and long-term carrier types were based on the observed heterogeneity in carriage duration for *S*. *aureus* [41, 42].

**Table 1:** Notation and descriptions of total population sizes.

Variable	Description	Value^a^	Reference
N	Total population size	165,300	[46, 47]
N_1_	Hospital population size	300	[46, 47]
N_2_	Community population size	165,000	[46, 47]
N_1,0–64_	Number of persons aged 0 to 64 years in hospital	174	[39]
N_1,>65_	Number of adults aged 65 years or older in hospital	126	[39]
N_2,0–64_	Number of persons aged 0 to 64 years in community	140,331	[39]
N_2,>65_	Number of adults aged 65 years or older in community	24,669	[39]

a. Total population sizes (N, N_1_, and N_2_) were the same across each of the five models. The age group variables applied only to the Age Group model, and the values shown here are for the “Age Group” parameter values given in Table 2.

**Table 2:** Parameter values and ranges used across models.

Param.	Description	Parameter values used in Models 1–5						Reference
		Age Group		Colonized Admission	Colonized LOS	Colonized Admission + LOS	Homogeneous Carriage	
p	Proportion of population that is >65 years old	0.15	0		0	0	0	[44]
q	Proportion of population that would be long-term carriers if they acquire carriage	0.20	0.20		0.20	0.20	0	[41, 45]
c	Ratio of community size to the number of occupied hospital beds	550:1	550:1		550:1	550:1	550:1	[46, 47]
β_1_	Transmissibility of carriers in hospital (1/days)	0.141^a^	0.111^a^		0.101^a^	0.068^a^	0.128^a^	Estimated
β_2_	Transmissibility of carriers in community (1/days)	0.011^a^	0.010^a^		0.010^a^	0.010^a^	0.003^a^	Estimated
σ	Proportional change in number of community contacts a >65 year old person makes compared to a 0–64 year old person	0.51	1		1	1	1	[48]
δ_1_	Assortativity of contacts between age groups in hospital	0.35^b^	0		0	0	0	[39]
δ_2_	Assortativity of contacts between age groups in community	0.167^c^	0		0	0	0	[48]
1/γ_1a_,	Average duration of colonization for short-term carriers in hospital and community (days)	30	30		30	30	278	[41, 45]
1/γ_2a_								
1/γ_1b_,	Average duration of colonization for long-term carriers in hospital and community (days)	365	365		365	365	278	[41, 45]
1/γ_2b_								
1/γ_S_	Average duration of symptoms for cases (days)	5	5		5	5	5	Assumed
1/r_U,0–64_	Average length of stay for 0–64 year old uncolonized individuals (days)	4.13^d^	4.5		4.466^e^	4.466^e^	4.5	[39]
r_>65_	Length of stay multiplier for >65 year old individuals	1.26	1		1	1	1	[39]
r_C_	Length of stay multiplier for length of stay for colonized individuals	1	1		1.22	1.22	1	[39]
r_S_	Discharge rate multiplier for symptomatic cases with respect to asymptomatically colonized individuals of that age group	0.5	0.5		0.5	0.5	0.5	Assumed
a_U,0–64_,	Admission rates by infection state and age group (1/days)	--^f^	--^f^		--^f^	--^f^	--^f^	Calculated
a_U,>65_,								
a_C,0–64_,								
a_C,>65_,								
a_S,0–64_,								
a_S,>65_								
ρ_>65_	Admission rate multiplier for >65 year olds	3.05^g^	1		1	1	1	[39]
ρ_C_	Admission rate multiplier for asymptomatically colonized individuals	1	1.3		1	1.3	1	[18]
ρ_S_	Admission rate multiplier for symptomatic individuals	2	2		2	2	2	Assumed
α_1,0–64_,	Rate of disease progression for 0–64 and >65 year olds in hospital (1/days)	0.009	0.009		0.009	0.009	0.009	[49]
α_1,>65_								
α_2,0–64_,	Rate of disease progression for 0–64 and >65 year olds in community (1/days)	α_1,0-64_/5, α_1,>65_/5	α_1,0-64_/5, α_1,>65_/5		α_1,0-64_/5, α_1,>65_/5	α_1,0-64_/5, α_1,>65_/5	α_1,0-64_/5, α_1,>65_/5	Assumed
α_2,>65_								
θ	Effectiveness of hospital transmission-based intervention ^h^	30%	30%		30%	30%	30%	Assumed
γ_int,0–64_,	Rate of decolonization due to intervention for colonized individuals in hospital (1/days)	0.035	0.035		0.035	0.035	0.035	Assumed
γ_int, >65_								

a. Transmissibility parameters were fitted using the least-squares method to obtain 3.4% prevalence in the hospital [29] and 1.5% prevalence in the community [40] for each model in the absence of any intervention.

b. To calculate the age-assortativity of patient contacts in the hospital using empirical data, we assumed that most transmission is within wards, and that children 0 to 17 years old and women giving birth would not mix with the adults aged 65 years and older. Using these assumptions, age-stratified national data on the total annual number of patient days from the Healthcare Cost and Utilization Project (HCUP) 2012 [39] and childbirth stay data from [50], we estimated that 40% of contacts that adults aged 65 years and older have in the hospital would be with persons aged 0 to 64 years old, whereas this fraction would be 61% if contacts occurred randomly between age groups. We adjusted δ_1_ until the percentage of contacts within and between age groups in the hospital matched our estimates.

c. Using symmetric contact matrices and all of the European survey data from POLYMOD [48], we calculated the proportion of individuals in each age group and the fraction of contacts within and between age groups. We adjusted δ_2_ so that the percentage of contacts by age group matched the POLYMOD data.

d. We adjusted discharge rates for the Age Group model so that the mean length of stay was kept at 4.5 days in line with the other models. For example, we used adults aged 65 years and older length of stay and total number of stays for from the Healthcare Cost and Utilization Project (HCUP) 2012 data [39]. 34.8% of discharges in HCUP were in the adults aged 65 years and older age group and this group had a mean LOS of 5.2 days. Therefore, we calculated that the persons aged 0 to 64 years old group must have a mean LOS of 4.13 days to produce an overall reported mean LOS of 4.5 days across both age groups.

e. In the Colonized LOS and Colonized Admission + LOS models, we assumed that the length of stay for asymptomatically colonized individuals was 1 day longer than that of uncolonized individuals. The length of stay for uncolonized individuals was adjusted to maintain a mean length of stay across all patients of 4.5 days.

f. We balanced the total number of admissions and discharges in order to keep the number of hospital patients constant. To do this, we calculated the persons aged 0 to 64 years old’s admission rate by dividing the number of daily hospital discharges by the community census, where the community census was the sum of all people in the community weighted by the values of the admission rate multipliers for adults aged 65 years and older, asymptomatic carriers, or symptomatic individuals relative to uncolonized persons 0 to 64 years old (ρ_>65_, ρ_C_, and ρ_S_ respectively). We then multiplied the admission rate for uncolonized persons 0 to 64 years old by the relevant admission rate multipliers to calculate the admission rates for the other groups.

g. After setting the length of stays for the Age Group model, ρ_>65_ was increased until the percentage of admissions that were adults aged 65 years and older in the simulations matched the estimate from national data of 34.8% of admissions being adults aged 65 years and older [39].

h. For example, an effectiveness value of 30% is consistent with a combined coverage of 50% and efficacy of 60% or coverage of 75% and efficacy of 40%.

Transmission could occur between patients in the hospital or between individuals in the community, and the infectiousness of those carrying the pathogen was allowed to differ between the hospital (β_1_) and community (β_2_). Colonized and symptomatic individuals in the same location were assumed to be equally infectious (i.e., had the same β_1_ or β_2_ values). However, contact rates could be assortative by age group, meaning individuals of one age group were more likely to contact members of the same age group than expected if contacts had occurred randomly. The degree of this assortativity could vary by location (δ_1_, δ_2_). Transmission from adults aged 65 or older in the community was reduced by a proportion (σ) to reflect the fewer overall number of social contacts of older adults compared to younger people [43]. Once colonized, individuals could become symptomatic at a rate α, and this rate could depend on age group and location. Asymptomatic carriers could clear colonization at rate γ_a_ for short-term carriers, or γ_b_ for long-term carriers. Symptomatic cases returned to the colonized state when they recovered from infection at rate γ_S_.

Admission (a) and discharge (r) rates could depend on age group and whether the person was uncolonized, colonized, or symptomatic. Admissions and discharges were balanced to keep hospital and community sizes constant. The lengths of stay in the hospital and rates of admission for colonized, symptomatic, or adults aged 65 or older could be equal to or higher than the rate of admission of uncolonized, persons aged 0 to 64 years according to the following admission rate multipliers: adults aged 65 or older (ρ_>65_), colonized (ρ_C_), or symptomatic (ρ_S_). These multipliers interacted multiplicatively for individuals with more than one risk factor for admission (e.g., adults aged 65 or older who were colonized were admitted at a rate ρ_>65_*ρ_C_ times that of uncolonized, persons aged 0 to 64 years).

We modeled two types of hospital interventions: one that decreased the infectiousness of carriers (hereafter termed “transmission-based intervention”) and another that increased the rate at which asymptomatic carriers cleared carriage (hereafter termed “decolonization”). These interventions can be thought of as simplifications of hand hygiene or use of barrier precautions (transmission-based interventions) and the use of antiseptic bathing and nasal decolonization (decolonization). To simulate the transmission-based intervention, the parameter θ proportionally reduced the infectiousness of asymptomatically colonized and symptomatic carriers in the hospital. The parameters γ_int,0–64_ and γ_int,>65_ represented the additional clearance rates due to hospital-based decolonization intervention. We assumed that the duration of symptoms would not be affected by the decolonization intervention.

### Sub-models for comparison

We used this general model to test the effects of different assumptions regarding asymptomatic carrier admission rates and lengths of stay on estimates of intervention impact. To help motivate the problem, we can consider the following hypothetical scenario. Suppose there are limited data suggesting a particular HAI intervention has been effective during outbreaks, but it has not been studied in endemic settings. Consequently, researchers want to assess the possible impact of the intervention in hospitals where the pathogen is endemic in order to inform decisions about the likely generalizability of empirical results. Ideally, the simulation should meet some basic criteria in order for the example to be plausible (e.g., the simulated prevalence of the pathogen in the hospital and community should match data from well-designed, large-scale screening studies). However, there are many different ways to make the simulation fit the observed data, and certain approaches could potentially skew results. Of particular concern are common modeling assumptions that asymptomatic carriers have higher rates of hospital admission or longer lengths of stay precisely because they are asymptomatically colonized, which have no biological basis. We analyzed a set of models to help inform modeling decisions in such a scenario.

We first devised parameter values to create five sub-models for comparison (Table 2). (1) Age Group model: we stratified the population into two age strata: persons 0 to 64 years old and adults aged 65 years and older, where older individuals had higher admission rates and longer lengths of stay in the hospital. No other model stratified the population into age groups. In the next three models, asymptomatically colonized individuals had (2) higher admission rates (Colonized Admission model), (3) longer length of stays (Colonized LOS model), or (4) both higher admission rates and longer lengths of stays (Colonized Admission + LOS model) compared to uncolonized individuals. We used the Age Group model as our base model against which we compared other model variants. Our main focus was on comparing Models 1–4. We also included a fifth model because of its widespread use (5) Homogeneous Carriage model: we assumed there were no differences in the average duration of carriage across the population. The average duration of carriage for the Homogeneous Carriage model was calculated as a weighted average of the mean durations of colonization used in the other models.

For each model, we fitted the hospital and community transmissibility parameters using the least-squares method to obtain 3.4% MRSA prevalence in the hospital and 1.5% prevalence in the community in the absence of interventions. Then, we ran the model to equilibrium and introduced either the transmission-based intervention, decolonization, or both. For each model, we compared the reproduction numbers (R) and percent of symptomatic cases averted by the interventions. We calculated the number of symptomatic cases averted by subtracting the number of symptomatic cases in a specific model with an intervention from the number of symptomatic cases for the same model without an intervention.

### Reproduction number calculation

A reproduction number (R) quantifies the spread of a pathogen in a population. We used the next-generation matrix method to calculate R [51], though we note that the reproduction numbers provided here do not have a standard R_0_ interpretation [52]. In addition to an overall R, we also calculated reproduction numbers that included only hospital transmission (R_hosp_) by setting community transmission to zero (i.e., β_2_ = 0). We similarly calculated a community reproduction number (R_comm_) for each model. Separate reproduction numbers for hospital and community transmission are commonly reported in the literature [12, 53–55] and help compare transmission dynamics in each setting.

### Sensitivity analyses

We also examined the effects of assumptions about colonized length of stay (r_C_) and colonized admission rate (ρ_C_) by manipulating the parameter ranges within a single model structure (Age group model). We divided each of the two multiplier parameters into ranges of values from 1 to 3, and then created combinations of each parameter value. For every combination of the two parameters, we used least squares to fit the hospital (β_1_) and community (β_2_) transmission rates to obtain 3.4% prevalence in the hospital [29] and 1.5% prevalence in the community [40] for each model in the absence of any intervention.

We then applied a transmission-based or decolonization intervention and calculated the number of symptomatic cases averted by each intervention. For each set of parameter values, we compared the number of cases averted to a base case when the multipliers were set to one (r_C_ = ρ_C_ = 1). This base case either represented the Age group model or the Homogeneous model; in both models, asymptomatic carriers have the same admission rate and length of stay as uncolonized individuals of the same age. We calculated the percent change in cases averted as the percentage of cases averted with multipliers equal to 1 minus the percentage of cases averted with multipliers equal to “x”, scaled by the percentage of cases averted with multipliers equal to 1. We visualized the results with a contoured heatmap and assessed differences across assumptions and treatments.

We used R (version 3.5.0) to create and analyze the model [56].

## Results

### Impact of assumptions that asymptomatic carriage affects admission rates or lengths of stay

In the pre-intervention period, the equilibrium prevalence of MRSA was 3.4% in the hospital and 1.5% in the community across all five models (Table 3). The admission prevalence was approximately 1.5% for the Age Group, Homogeneous, and Colonized LOS models, whereas admission prevalence was 1.96% for both models that assumed that colonized individuals were more likely to be admitted to the hospital than uncolonized individuals (i.e., Colonized Admission, Colonized Admission + LOS). The rate of carriage acquisition in the hospital ranged from 2.33/1,000 uncolonized patient-days (Colonized Admission + LOS model) to 4.79/1,000 uncolonized patient-days (Age Group model). As expected, the Homogeneous model had the lowest R (1.021), while R was 1.046 for all other models (Table 3).

**Table 3:** Impact of model assumptions on intervention effects using baseline parameters^a^.

Metric	Age Group	Homogeneous	Colonized Admission	Colonized LOS	Colonized Admission + LOS
R	1.046	1.021	1.046	1.046	1.046
R_hosp_	0.624	0.637	0.477	0.513	0.35
R_comm_	1.000	0.873	1.003	1.007	1.019
β_1_	0.1412	0.1275	0.1109	0.1014	0.0684
β_2_	0.011	0.0031	0.0103	0.0103	0.0104
Pre-intervention hospital colonization prevalence	3.40%	3.40%	3.40%	3.40%	3.40%
Pre-intervention community colonization prevalence	1.50%	1.50%	1.50%	1.50%	1.50%
Pre-intervention hospital admission prevalence	1.52%	1.51%	1.96%	1.51%	1.96%
Pre-intervention hospital admission prevalence for persons aged 0 to 64 years old	1.51%	--	--	--	--
Pre-intervention hospital admission prevalence for adults aged 65 years and older	1.54%	--	--	--	--
Hospital rate of carriage acquisition (per 1000 uncolonized patient-days)	4.79	4.33	3.77	3.45	2.33
Percent of colonized patients decolonized before discharge by decolonization intervention^b^	14.3%	13.8%	13.8%	16.3%	16.3%
Percent of symptomatic cases averted^c^ (decolonization intervention, γ_int,0–64_ = γ_int,>65_ = 0.035)	8.9%	17.8%	9.3%	9.2%	9.1%
Percent of symptomatic cases averted^c^ (transmission-based intervention effectiveness (θ = 30%)	7.3%	18.8%	5.6%	5.3%	3.1%
Percent of symptomatic cases averted^c^ (both interventions simultaneously, θ = 30% and γ_int,0–64_ = γ_int,>65_ = 0.035)	13.0%	27.3%	12.7%	12.1%	10.9%

a. The parameters used in these simulations are listed in the models’ respective columns in Table 2.

b. Percent of colonized patients decolonized before discharge is the probability that a colonized individual in the hospital reverted to being uncolonized before being discharged because of the decolonization intervention. For the Age Group model, the discharge rate was weighted by the relative proportions of persons aged 0 to 64 years old and adults aged 65 years and older in the hospital.

c. We combined hospital and community cases in calculations of the percent of symptomatic cases averted.

**Fig 2:**
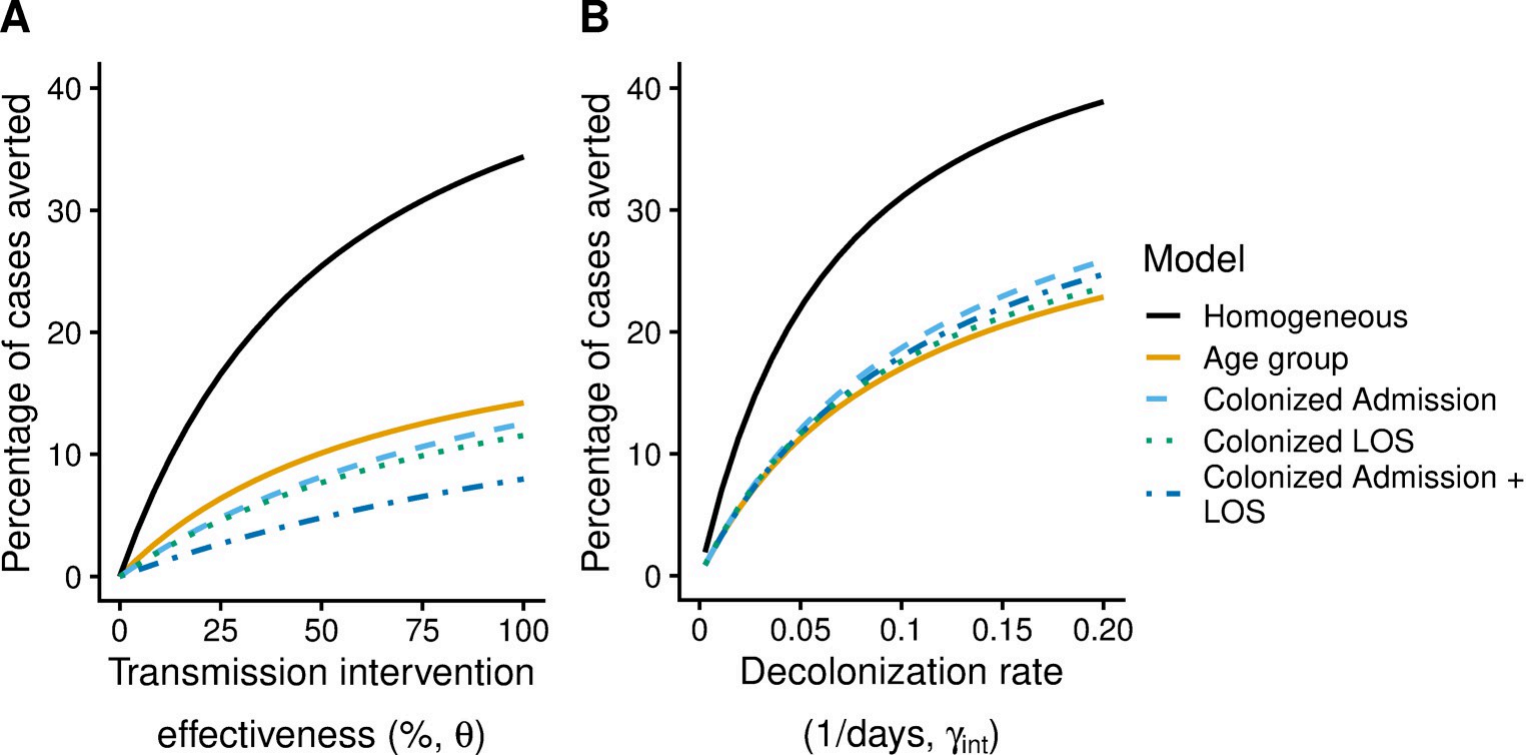
Impact of model assumptions on the percentage of symptomatic cases averted by hospital interventions. Percent of symptomatic cases averted after 5 years of a hospital: (A) transmission-based intervention that reduced the infectiousness of carriers (both asymptomatically colonized and symptomatic cases) or (B) decolonization intervention that reduced the duration of carriage for asymptomatically colonized patients. The y-axis shows the percent of symptomatic cases averted over 5 years in the entire population under simulated interventions that began with the pathogen at equilibrium. We combined hospital and community cases (both asymptomatic and symptomatic), and calculated that 8,223 cases occurred over 5 years in each model in the absence of any intervention. In (A), the transmission-based intervention effectiveness was the proportional reduction in the infectiousness of all MRSA patients (i.e., proportional reduction in β_1_). In (B), the intervention’s decolonization rate was the average increase in the rate of loss of asymptomatic carriage in the hospital. Colors show the different models (*Age Group* = solid black; *Colonized Admission* = dashed light blue; *Colonized LOS* = dot-dash dark blue; *Colonized Admission* + *LOS* = dotted green; *Homogeneous* = solid yellow). See Table 2 for parameter values used for each model.

The effect of the different model assumptions on the estimated impact of a hospital intervention depended on the type of intervention (Fig 2, Table 3). In the absence of any interventions, 8,223 cases occurred over 5 years in each model. For the transmission-based intervention, the percentage of symptomatic cases averted was highest for the Homogeneous Model, followed by the following models in order: Age Group, Colonized Admission, Colonized LOS, and Colonized Admission + LOS. (Fig 2A). The differences in intervention impact between the models occurred across a wide range of transmission-based intervention effectiveness values (Fig 2).

For the decolonization intervention, the percent of symptomatic cases averted was again largest for the Homogeneous Carriage model, but was similar across the other models until the rate of decolonization became high (Fig 2B, Table 3). Results for scenarios where both interventions were applied concomitantly were in line with findings for each intervention applied separately (S1 Fig).

### Analysis of model parameters’ influence on how carrier assumptions change model output

**Fig 3:**
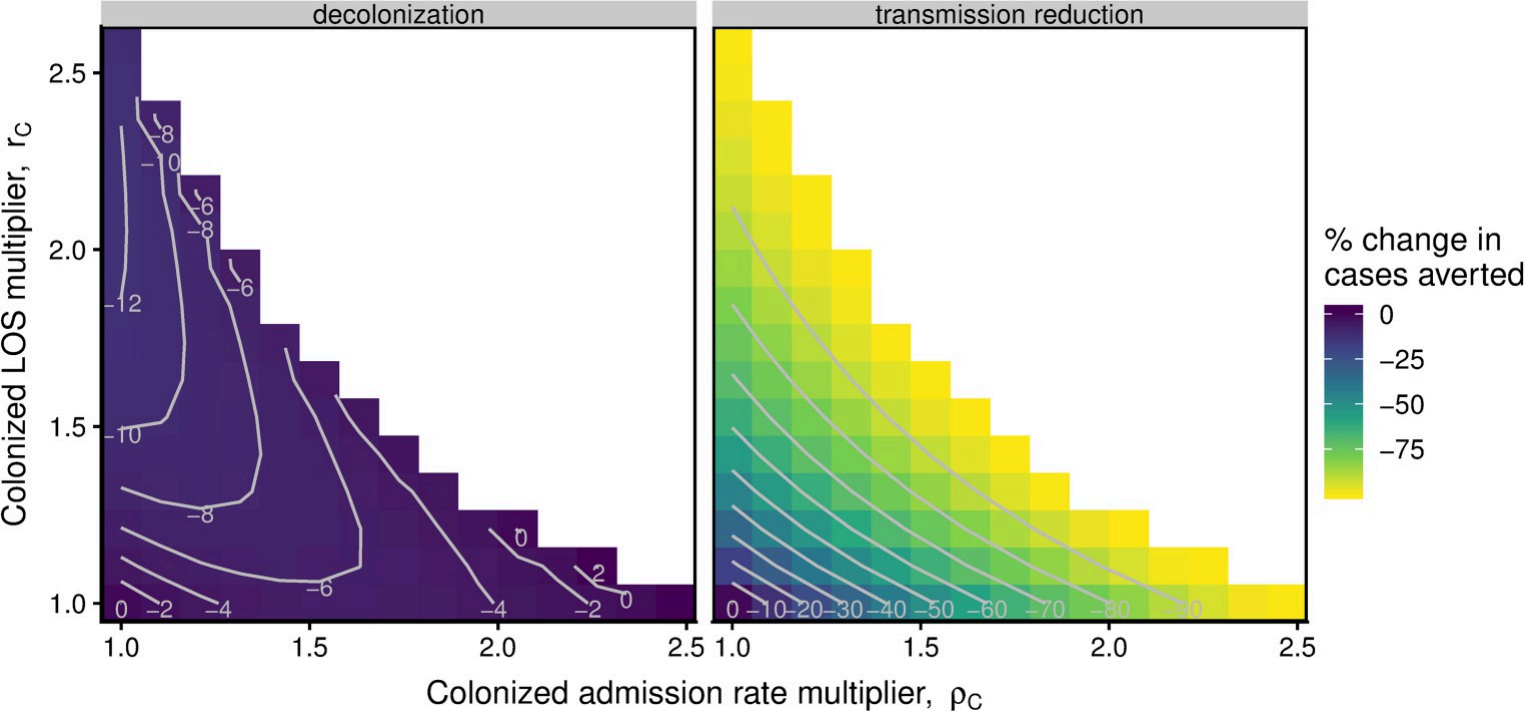
Sensitivity of number of symptomatic cases averted to uncertainty in parameters for increased length of stay or admission rate for asymptomatic individuals. The percent change in number of symptomatic cases averted in the Age Group Model when increasing the colonized admission rate multiplier (ρ_C_) and/or the colonized length of stay multiplier (r_C_) compared to the base case (i.e., r_C_ = ρ_C_ = 1). Colors and contour lines denote the percentage change in cases averted compared to the base case. For each combination of values for the r_C_ and ρ_C_ multipliers, the transmission parameters were refit to achieve an equilibrium prevalence of 3.4% colonized individuals in the hospital and 1.5% in the community. We ran models to equilibrium and then implemented a decolonization intervention (γ_int_ = 0.035) or a transmission-based intervention (θ = 30%).

All else being equal, including assumptions where asymptomatically colonized individuals had increased length of stay or admission rates because of their colonization status heavily affected estimates of the impact of a transmission-based intervention but had modest effects for a decolonization intervention (Fig 3). Both multipliers had similarly strong effects for the transmission-based intervention. However, the length of stay multiplier (r_C_) had a stronger relative impact than the admission rate multiplier for the decolonization intervention. Higher values of length of stay multiplier or colonized admission rates generally led to greater differences in the number of cases averted. However, when using the homogeneous model as the baseline case, results were more consistent across treatment types (S2 Fig).

## Discussion

We showed that assuming asymptomatic carriers have higher rates of hospital admission or longer lengths of stay can bias simulations of the impact of hospital-based HAI interventions designed to prevent spread of healthcare-associated pathogens, especially for transmission-based interventions (e.g., enhanced infection control efforts). These assumptions are typically included as if asymptomatic carriers had more healthcare exposure precisely because they were asymptomatically colonized [17–19, 21–35]. However, confounding and reverse causation are more likely explanations for observed differences in admission rates and lengths of stay [36].

In our main example (Fig 2), the carrier assumptions produced larger differences across models in the impact of a transmission-based intervention than for a decolonization intervention. Compared to the Age Group model, fewer cases were predicted to be averted by a transmission-based intervention for models where colonized individuals had longer lengths of stay or higher admission rates. The most likely explanation is that when we assumed higher admission rates or longer lengths of stay for colonized individuals, then hospital transmissibility did not need to be as high to reach the same equilibrium hospital prevalence compared to the Age Group model, and therefore the community became relatively more important for pathogen transmission. In other words, the transmission-based intervention, which proportionally reduced transmissibility in the hospital (β_1_), prevented fewer transmissions in the Colonized Admission, Colonized LOS, and Colonized Admission + LOS models than in the Age Group model because the latter had the highest value of β_1_ to begin with, the highest R_hosp_ value, and therefore the largest contribution of hospital transmission to the overall R value. In contrast, assuming homogeneous lengths of carriage resulted in much higher numbers of transmissions and cases averted than in the Age Group model because the Homogeneous Carriage model had a lower R and transmission in the Homogeneous Carriage model was even more heavily weighted towards transmission in the hospital. On the other hand, decolonization treatments had smaller differences across models, possibly because the number of colonized individuals in the hospital was always the same so only a certain number could be decolonized. For combinations of decolonization and transmission-based interventions, comparisons across models could give similar results depending on the value of each intervention parameter (S1 Fig).

A potentially valid reason for parameterizing higher admission rates or longer lengths of stay for individuals in model compartments containing asymptomatic carriers is when symptomatic and colonized individuals are pooled together. In this case, the combined compartments could be parameterized using the weighted average of parameter values for colonized and symptomatic individuals [57]. However, such averaged parameter values would need to change in response to an intervention that affected the ratio of asymptomatic versus symptomatic carriers, and it could be difficult to calculate what the new ratio and parameter values should be without representing asymptomatic and symptomatic carriers separately. As with analyses that use standard statistical models (e.g., case-control studies analyzed with logistic regression), we recommend that the choice of which variables to include in a transmission model should be guided by the research question as well as subject matter considerations [58].

Additionally, we assessed the influence of these assumptions over a range of potential values. In general, the stronger the assumption about asymptomatically colonized individuals and their propensity to stay in the hospital, the more one might miscalculate the impact of an intervention. In our simulations, even small changes in the multipliers led to substantial differences in the model results. However, the type of intervention is still important: we observed different results for an intervention to reduce transmission compared to an intervention that decolonized patients, likely because decolonization acts more through recovery and transmission-based interventions directly reduces transmissibility.

Our analyses have several limitations. For example, we omitted antibiotics, competition from other pathogen strains (*e*.*g* methicillin-sensitive *Staphylococcus aureus*) or species (*e*.*g*. *Clostridioides difficile*), and impact of patient transfers to or from additional healthcare facilities healthcare facilities, each of which could have affected the magnitude of our results. However, we expect that the same issues would remain if such factors were included because assuming that asymptomatic carriers are more likely to be admitted to hospital or have longer lengths of stay would still have no biological basis and would continue to artificially inflate pathogen prevalence in the hospital compared to a more appropriately structured model (e.g., finer age stratification, higher admission rates for recently discharged patients or nursing home residents). We also expect similar problems to occur if pathogen incidence data were used for fitting instead of prevalence data. Lastly, including higher rates of hospitalization and longer lengths of stay for individuals recently discharged from hospital compared to those who have not been hospitalized recently would have improved the accuracy of the model. However, such model complexity was not required to illustrate the problem, and therefore doing so would have made our example more difficult without clearly providing a commensurate benefit. In future work, it could be useful to include such additional complexity and assess how well different models can simultaneously fit contemporaneous estimates of community prevalence, admission prevalence, hospital prevalence, and/or hospital incidence, which could also help discriminate between simulations in addition to using subject matter knowledge to guide modeling decisions.

In our Age Group model, we stratified the population into groups of younger and older individuals, and each age group had different admission rates and lengths of stay. However, we do not expect that this would result in the same problems as including changes in admission rates and lengths of stay being a direct consequence of being an asymptomatic carrier. Age is a proxy for factors that more directly affect admission rates and lengths of stay (e.g., being diagnosed with a health condition, insurance status). Such factors are unlikely to change concurrently with the gain and loss of colonization, especially after having included additional states in the model (e.g., hospitalization, which is correlated with health status and the risk of acquiring a pathogen), and there are no interventions that affect date of birth. Therefore, we believe that the Age Group model is more realistic compared to the other models and it was useful as a simple base case against which to compare the other modeling assumptions. Building on this framework, additional risk groups could be considered for inclusion as necessary. For example, male gender, recent hospitalization, and residence in a nursing home are risk factors for *S*. *aureus* colonization and bacteremia due to an *S*. *aureus* infection [59–62], and each could be considered for inclusion as additional risk groups.

A simple model cannot capture every possible factor, but our simplified model helped illustrate the central problem associated with attributing different admission rates or lengths of stay to asymptomatic carriers without a clear biological basis for doing so. Our results show that such assumptions can greatly affect estimates of the impact of interventions, and therefore should be justified. We have included our model code, which can be adapted to represent other HAI pathogens and risk groups by changing parameter values or adapting the model structure. While the pathogen parameters in our main example were based on MRSA, our conclusions are broadly applicable because assuming that asymptomatic carriers are more likely to be admitted to hospital or have longer lengths of stay generally have no biological basis.

## Conclusions

It is important to assess how modeling assumptions influence estimates of intervention impact. We have shown that common assumptions regarding how colonized individuals are included in simulations can heavily bias estimates of the impact of transmission interruption and to a lesser extent decolonization interventions. Modelers ought to consider whether data in favor of such assumptions could also be biased, how including such assumptions can affect model results, and if a more complex model (e.g., that includes ≥1 risk factor) is necessary to address specific research aims. Decision-makers should also critically evaluate the biological assumptions underpinning parameter values used in models that inform policy.

## Supporting information

S1 Appendix Model equations. (DOCX)

S1 Fig Heatmap of the percentage of cases averted for simultaneous decolonization and transmission-based interventions. Lighter colors show higher percentages of cases averted, and the range of decolonization and transmission-based intervention effectiveness is the same as in Fig 2.
(TIF)

S2 Fig Sensitivity of number of symptomatic cases averted to uncertainty in parameters for increased length of stay or admission rate for asymptomatic individuals. The percent change in number of symptomatic cases averted in the Homogeneous Model when run to equilibrium and then either a decolonization intervention (γ_int_ = 0.035) or a transmission-based intervention (θ = 30%) implemented.
(TIF)

S1 Table State variable table with description of notation. (DOCX)
