# Supplementary material for: Improving mathematical modeling of interventions to prevent healthcare-associated infections by interrupting transmission or pathogens: How common modeling assumptions about colonized individuals impact intervention effectiveness estimates
Source: PLoS One. 2022 Feb 28;17(2):e0264344. doi: 10.1371/journal.pone.0264344 (PMC8884501; doi:10.1371/journal.pone.0264344)
Supplement: S1 Appendix — (DOCX) [file pone.0264344.s001.docx]

**S1 Appendix: Model equations**

Ordinary differential equations for a model with compartments for each combination of (i) Uncolonized (U), Colonized (C), or Symptomatic (S); (ii) young (0-64 years) or old (>65 years); (iii) short-term carrier (a) or long-term carrier (b); and (iv) in a hospital (1) or community (2):

$\dot{U}_{1b,0-64}=-\lambda_{1,0-64}U_{1b,0-64}+\left( \gamma_{1b}+\gamma_{int,0-64} \right)C_{1b,0-64}+a_{U,0-64}U_{2b,0-64}-r_{U,0-64}U_{1b,0-64}$ (1)

$\dot{U}_{1a,0-64}=-\lambda_{1,0-64}U_{1a,0-64}+\left( \gamma_{1a}+\gamma_{int,0-64} \right)C_{1a,0-64}+a_{U,0-64}U_{2a,0-64}-r_{U,0-64}U_{1a,0-64}$ (2)

$\dot{U}_{1b,>65}=-\lambda_{1,>65}U_{1b,>65}+\left( \gamma_{1b}+\gamma_{int,>65} \right)C_{1b,>65}+a_{U,>65}U_{2b,>65}-r_{U,>65}U_{1b,>65}$ (3)

$\dot{U}_{1a,>65}=-\lambda_{1,>65}U_{1a,>65}+\left( \gamma_{1a}+\gamma_{int,>65} \right)C_{1a,>65}+a_{U,>65}U_{2a,>65}-r_{U,>65}U_{1a,>65}$ (4)

$\dot{U}_{2b,0-64}=-\lambda_{2,0-64}U_{2b,0-64}+\gamma_{2b}C_{2b,0-64}-a_{U,0-64}U_{2b,0-64}+r_{U,0-64}U_{1b,0-64}$ (5)

$\dot{U}_{2a,0-64}=-\lambda_{2,0-64}U_{2a,0-64}+\gamma_{2a}C_{2a,0-64}-a_{U,0-64}U_{2a,0-64}+r_{U,0-64}U_{1a,0-64}$ (6)

$\dot{U}_{2b,>65}=-\lambda_{2,>65}U_{2b,>65}+\gamma_{2b}C_{2b,>65}-a_{U,>65}U_{2b,>65}+r_{U,>65}U_{1b,>65}$ (7)

$\dot{U}_{2a,>65}=-\lambda_{2,>65}U_{2a,>65}+\gamma_{2a}C_{2a,>65}-a_{U,>65}U_{2a,>65}+r_{U,>65}U_{1a,>65}$ (8)

$\dot{C}_{1b,0-64}=\lambda_{1,0-64}U_{1b,0-64}-\left( \gamma_{1b}+\gamma_{int,0-64} \right)C_{1b,0-64}+a_{C,0-64}C_{2b,0-64}-r_{C,0-64}C_{1b,0-64}-\alpha_{1,0-64}C_{1b,0-64}+\gamma_{S}S_{1b,0-64}$ (9)

$\dot{C}_{1a,0-64}=\lambda_{1,0-64}U_{1a,0-64}-\left( \gamma_{1a}+\gamma_{int,0-64} \right)C_{1a,0-64}+a_{C,0-64}C_{2a,0-64}-r_{C,0-64}C_{1a,0-64}-\alpha_{1,0-64}C_{1a,0-64}+\gamma_{S}S_{1a,0-64}$ (10)

$\dot{C}_{1b,>65}=\lambda_{1,>65}U_{1b,>65}-\left( \gamma_{1b}+\gamma_{int,>65} \right)C_{1b,>65}+a_{C,>65}C_{2b,>65}-r_{C,>65}C_{1b,>65}-\alpha_{1,>65}C_{1b,>65}+\gamma_{S}S_{1b,>65}$ (11)

$\dot{C}_{1a,>65}=\lambda_{1,>65}U_{1a,>65}-\left( \gamma_{1a}+\gamma_{int,>65} \right)C_{1a,>65}+a_{C,>65}C_{2a,>65}-r_{C,>65}C_{1a,>65}-\alpha_{1,>65}C_{1a,>65}+\gamma_{S}S_{1a,>65}$ (12)

$\dot{C}_{2b,0-64}=\lambda_{2,0-64}U_{2b,0-64}-\gamma_{2b}C_{2b,0-64}-a_{C,0-64}C_{2b,0-64}+r_{C,0-64}C_{1b,0-64}-\alpha_{2,0-64}C_{2b,0-64}+\gamma_{S}S_{2b,0-64}$ (13)

$\dot{C}_{2a,0-64}=\lambda_{2,0-64}U_{2a,0-64}-\gamma_{2a}C_{2a,0-64}-a_{C,0-64}C_{2a,0-64}+r_{C,0-64}C_{1a,0-64}-\alpha_{2,0-64}C_{2a,0-64}+\gamma_{S}S_{2a,0-64}$ (14)

$\dot{C}_{2b,>65}=\lambda_{2,>65}U_{2b,>65}-\gamma_{2b}C_{2b,>65}-a_{C,>65}C_{2b,>65}+r_{C,>65}C_{1b,>65}-\alpha_{2,>65}C_{2b,>65}+\gamma_{S}S_{2b,>65}$ (15)

$\dot{C}_{2a,>65}=\lambda_{2,>65}U_{2a,>65}-\gamma_{2a}C_{2a,>65}-a_{C,>65}C_{2a,>65}+r_{C,>65}C_{1a,>65}-\alpha_{2,>65}C_{2a,>65}+\gamma_{S}S_{2a,>65}$ (16)

$\dot{S}_{1b,0-64}=\alpha_{1,0-64}C_{1b,0-64}-\gamma_{S}S_{1b,0-64}+a_{S,0-64}S_{2b,0-64}-r_{S,0-64}S_{1b,0-64}$ (17)

$\dot{S}_{1a,0-64}=\alpha_{1,0-64}C_{1a,0-64}-\gamma_{S}S_{1a,0-64}+a_{S,0-64}S_{2a,0-64}-r_{S,0-64}S_{1a,0-64}$ (18)

$\dot{S}_{1b,>65}=\alpha_{1,>65}C_{1b,>65}-\gamma_{S}S_{1b,>65}+a_{S,>65}S_{2b,>65}-r_{S,>65}S_{1b,>65}$ (19)

$\dot{S}_{1a,>65}=\alpha_{1,>65}C_{1a,>65}-\gamma_{S}S_{1a,>65}+a_{S,>65}S_{2a,>65}-r_{S,>65}S_{1a,>65}$ (20)

$\dot{S}_{2b,0-64}=\alpha_{2,0-64}C_{2b,0-64}-\gamma_{S}S_{2b,0-64}-a_{S,0-64}S_{2b,0-64}+r_{S,0-64}S_{1b,0-64}$ (21)

$\dot{S}_{2a,0-64}=\alpha_{2,0-64}C_{2a,0-64}-\gamma_{S}S_{2a,0-64}-a_{S,0-64}S_{2a,0-64}+r_{S,0-64}S_{1a,0-64}$ (22)

$\dot{S}_{2b,>65}=\alpha_{2,>65}C_{2b,>65}-\gamma_{S}S_{2b,>65}-a_{S,>65}S_{2b,>65}+r_{S,>65}S_{1b,>65}$ (23)

$\dot{S}_{2a,>65}=\alpha_{2,>65}C_{2a,>65}-\gamma_{S}S_{2a,>65}-a_{S,>65}S_{2a,>65}+r_{S,>65}S_{1a,>65}$ (24)

Force of infection equations to young (0-64 years) or old (>65 years) susceptible populations in a hospital (1) or the community (2), with transmission-based interventions in the hospital of effectiveness (θ) and assortativity of contacts between age groups (δ) in each location:

$\lambda_{1,0-64}=\beta_{1}\left( 1-\theta\right)\left( \left( 1-\delta_{1} \right)\frac{N_{1,>65}}{N_{1}} \right)\frac{\left( C_{1b,>65}+C_{1a,>65}+S_{1b,>65}+S_{1a,>65} \right)}{N_{1,>65}}+\beta_{1}\left( 1-\theta\right)\left( \delta_{1}+\left( 1-\delta_{1} \right)\frac{N_{1,0-64}}{N_{1}} \right)\frac{\left( C_{1b,0-64}+C_{1a,0-64}+S_{1b,0-64}+S_{1a,0-64} \right)}{N_{1,0-64}}$

$\lambda_{1,>65}=\beta_{1}\left( 1-\theta\right)\left( \left( 1-\delta_{1} \right)\frac{N_{1,0-64}}{N_{1}} \right)\frac{\left( C_{1b,0-64}+C_{1a,0-64}+S_{1b,0-64}+S_{1a,0-64} \right)}{N_{1,0-64}}+\beta_{1}\left( 1-\theta\right)\left( \delta_{1}+\left( 1-\delta_{1} \right)\frac{N_{1,>65}}{N_{1}} \right)\frac{\left( C_{1b,>65}+C_{1a,>65}+S_{1b,>65}+S_{1a,>65} \right)}{N_{1,>65}}$

$\lambda_{2,0-64}={\sigma\beta}_{2}\left( \left( 1-\delta_{2} \right)\frac{N_{2,>65}}{N_{2}} \right)\frac{\left( C_{2b,>65}+C_{2a,>65}+S_{2b,>65}+S_{2a,>65} \right)}{N_{2,>65}}+\beta_{2}\left( \delta_{2}+\left( 1-\delta_{2} \right)\frac{N_{2,0-64}}{N_{2}} \right)\frac{\left( C_{2b,0-64}+C_{2a,0-64}+S_{2b,0-64}+S_{2a,0-64} \right)}{N_{2,0-64}}$

$\lambda_{2,>65}=\beta_{2}\left( \left( 1-\delta_{2} \right)\frac{N_{2,0-64}}{N_{2}} \right)\frac{\left( C_{2b,0-64}+C_{2a,0-64}+S_{2b,0-64}+S_{2a,0-64} \right)}{N_{2,0-64}}+{\sigma\beta}_{2}\left( \delta_{2}+\left( 1-\delta_{2} \right)\frac{N_{2,>65}}{N_{2}} \right)\frac{\left( C_{2b,>65}+C_{2a,>65}+S_{2b,>65}+S_{2a,>65} \right)}{N_{2,>65}}$

Discharge rates for combinations of Uncolonized (U), Colonized (C), or Symptomatic (S), young (0-64 years), and old (>65 years) populations calculated using multipliers that increase length of stay (r_>65_ and r_C_) and multipliers that decrease the discharge rate itself (r_s_):

$r_{U,>65}=\frac{r_{U,0-64}}{r_{>65}}$

$r_{C,0-64}=\frac{r_{U,0-64}}{r_{C}}$

$r_{C,>65}=\frac{r_{U,0-64}}{(r_{C})(r_{>65})}$

$r_{S,0-64}=r_{S}\frac{r_{U,0-64}}{r_{C}}$

$r_{S,>65}=r_{S}\frac{r_{U,0-64}}{(r_{C})(r_{>65})}$

**Reproduction number (R) calculation**

We calculated reproduction numbers using the next-generation matrix method [51]. Here, we generated a transmission matrix (**T**) and a transition matrix (**Σ**) for each model. The transmission matrix details the production of new colonized individuals through transmission, while the transition matrix describes the movement of individuals between compartments, through admission, discharge, and disease progression, recovery, and loss of carriage. The next-generation matrix was calculated by multiplying the inverse of the transition matrix by the negative of the transmission matrix (**-T** * **Σ^-1^**). R was calculated as the dominant eigenvalue of the next-generation matrix. A reproduction number for the average number of carriers arising from an average infectious individual over the length of their infectious period spent in the hospital (R_hosp_) was calculated as above with replacement of the transmission terms for the community with zeros (i.e., by setting β_2_ = 0). A reproduction number for the average number of carriers arising from an average infectious individual over the length of their infectious period spent in the community (R_comm_) was calculated similarly.

**Software packages for data analysis**

The model was analyzed using packages *deSolve* (1.21), *rootSolve* (1.7), *socialmixr* (0.1.2), and results were visualized with the packages *ggplot2* (3.3.0) and *cowplot* (0.9.3) in R version 3.5.0 [52].
