# Supplementary figures and images for: Improving mathematical modeling of interventions to prevent healthcare-associated infections by interrupting transmission or pathogens: How common modeling assumptions about colonized individuals impact intervention effectiveness estimates
Source: PLoS One. 2022 Feb 28;17(2):e0264344. doi: 10.1371/journal.pone.0264344 (PMC8884501; doi:10.1371/journal.pone.0264344)

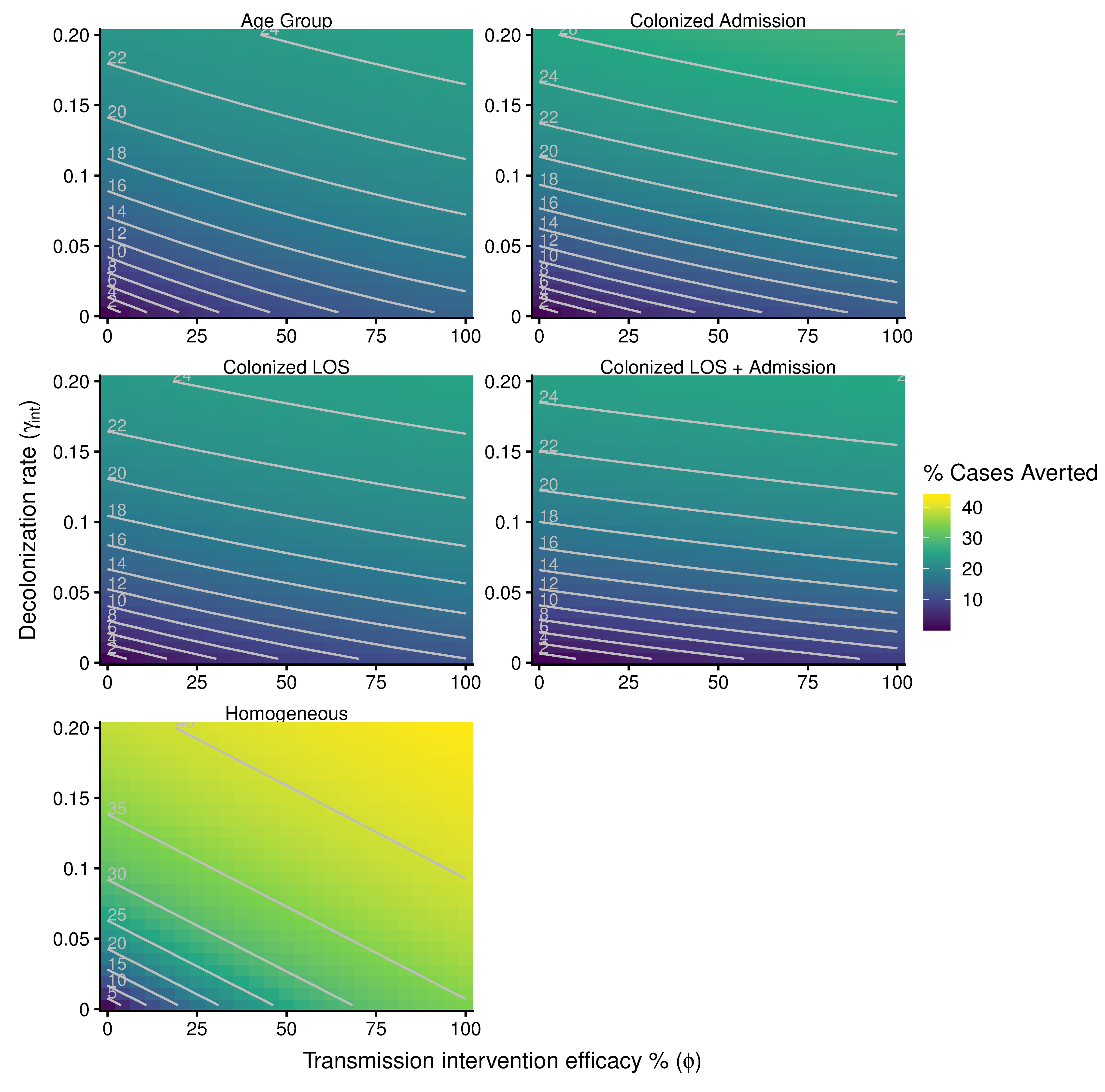

Supplement: S1 Fig — Lighter colors show higher percentages of cases averted, and the range of decolonization and transmission-based intervention effectiveness is the same as in Fig 2. (TIF) [file pone.0264344.s002.tif]

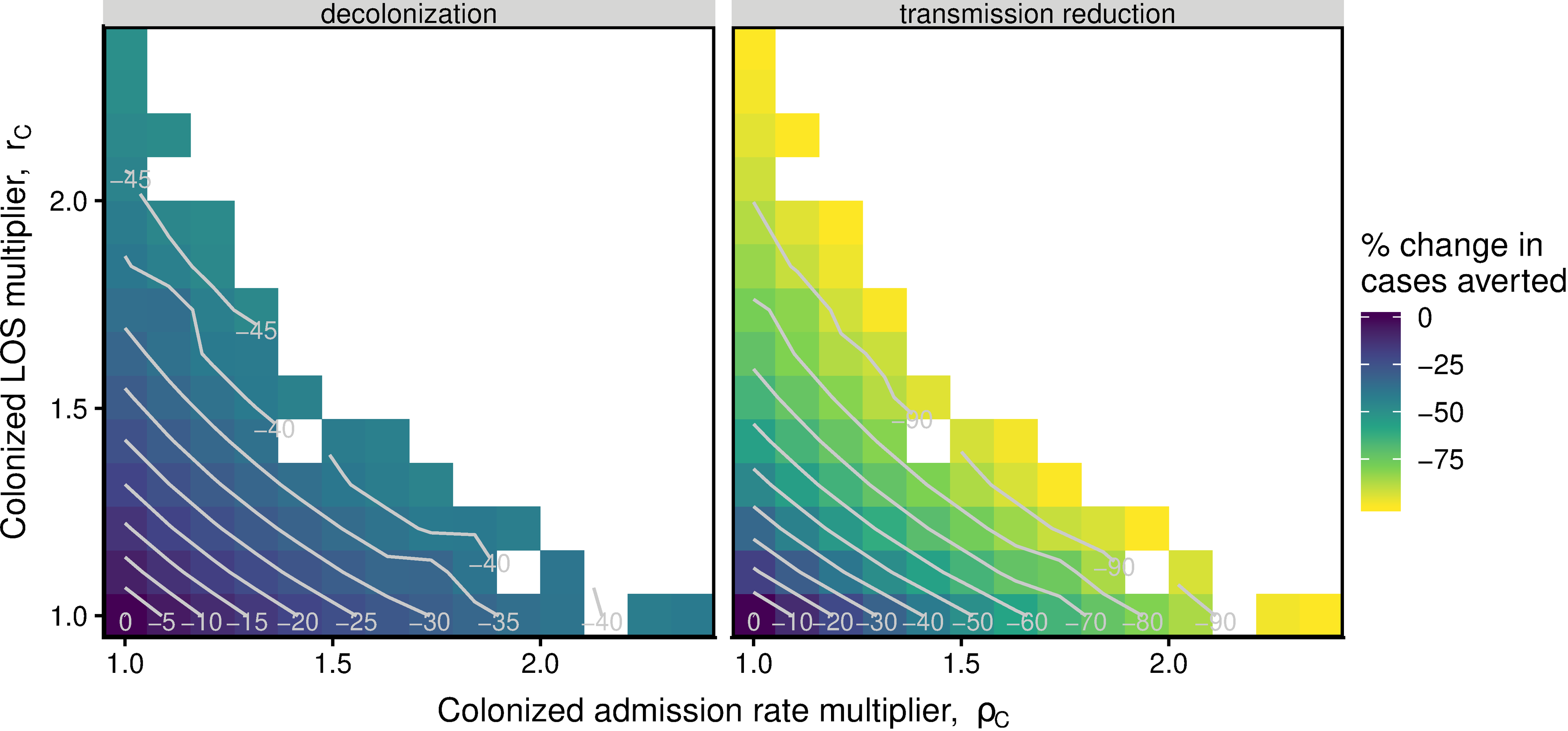

Supplement: S2 Fig — The percent change in number of symptomatic cases averted in the Homogeneous Model when run to equilibrium and then either a decolonization intervention (γint = 0.035) or a transmission-based intervention (θ = 30%) implemented. (TIF) [file pone.0264344.s003.tif]
