# Supplementary material for: Improving mathematical modeling of interventions to prevent healthcare-associated infections by interrupting transmission or pathogens: How common modeling assumptions about colonized individuals impact intervention effectiveness estimates
Source: PLoS One. 2022 Feb 28;17(2):e0264344. doi: 10.1371/journal.pone.0264344 (PMC8884501; doi:10.1371/journal.pone.0264344)
Supplement: S1 Table — (DOCX) [file pone.0264344.s004.docx]

**Table S1.** State variable table with description of notation

| Variable | Description | Value^a^ |
| --- | --- | --- |
| N | Total population size | 165,300 |
| N_1_ | Hospital population size | ­300 |
| N_2_ | Community population size | 165,000 |
| N_1,0-64_ | Number of 0-64 year olds in hospital | 174 |
| N_1,>65_ | Number of >65 year olds in hospital | 126 |
| N_2,0-64_ | Number of 0-64 year olds in community | 140,331 |
| N_2,>65_ | Number of >65 year olds in community | 24,669 |
| U_xxx_ | Uncolonized population^b^ | -- |
| C_xxx_ | Colonized (asymptomatic carriers) population | -- |
| S_xxx_ | Symptomatic carriers population | -- |
| X_xx,0-64_ | Number of 0-64 year olds in population | -- |
| X_xx,>65_ | Number of >65 year olds in population | -- |
| X_1xx_ | Hospital population size | -- |
| X_2xx_ | Community population size | -- |
| X_xax_ | Short-term carrier when colonized | -- |
| X_xbx_ | Long-term carrier when colonized | -- |

1. The total population size (N) was the same across each model. However, the total number of people in the hospital or community (N_1_, N_2_) could change as the ratio of community size to the number of occupied hospital beds was varied (i.e., parameter ‘c’ in Table 2). The age group variables applied only to the Age Group model, and the values show here are for the ‘Age Group’ parameter values given in Table 2.
2. “X” is a placeholder that includes all variations for that position (e.g., U_xxx_ includes all uncolonized individuals, regardless of age group, location, or carrier type).
